# Prevalence and severity of abscesses and cellulitis, and their associations with other health outcomes, in a community-based study of people who inject drugs in London, UK

**DOI:** 10.1371/journal.pone.0235350

**Published:** 2020-07-14

**Authors:** Talen Wright, Vivian Hope, Daniel Ciccarone, Dan Lewer, Jenny Scott, Magdalena Harris

**Affiliations:** 1 Department of Public Health, Environments & Society, London School of Hygiene & Tropical Medicine, London, United Kingdom; 2 Public Health Institute, Liverpool John Moores University, Liverpool, United Kingdom; 3 Department of Family and Community Medicine, University of California, San Francisco, San Francisco, CA, United States of America; 4 Institute of Epidemiology and Healthcare, University College London, London, United Kingdom; 5 Department of Pharmacy and Pharmacology, University of Bath, Claverton Down, Bath, United Kingdom; University of New South Wales, AUSTRALIA

## Abstract

**Background:**

Skin and soft tissue infections (SSTI) are a common but preventable cause of morbidity and mortality among people who inject drugs (PWID). They can be severe, and hospitalisations of PWID with SSTI are rising. The most common SSTI presentations are abscesses and cellulitis.

**Methods:**

We used data from Care & Prevent, a cross-sectional community survey of PWID in London. We reported the lifetime prevalence of SSTI, severity of infections, key risk factors, and associated sequelae. Pictorial questions were used to assess SSTI severity.

**Results:**

We recruited 455 PWID. SSTI lifetime prevalence was high: 64% reported an abscess and/or cellulitis. Over one-third (37%) reported a severe infection, 137 (47%) reported hospitalisation. SSTIrisk factors were: aged 35+ years, injecting once or more times a day, subcutaneous or intra-muscular injections, and making four or more attempts to achieve an injection. Those who reported having other health conditions were at higher odds of having an abscess or cellulitis, with risk tending to increase with number of reported conditions. Half (46%) employed self-care for their worst SSTI, and 43% waited for ten or more days before seeking medical care or not seeking medical care at all.

**Conclusions:**

Abscess and cellulitis are very common among PWID in London. We corroborate findings indicating SSTIs are associated with risks, e.g. venous access problems, as well as other co-morbid conditions: septicaemia, endocarditis, DVT, and kidney disease. These co-morbidities may impact SSTIs severity and outcomes. Delayed healthcare seeking potentially exacerbates infection severity, which in turn increases poorer health outcomes and complications.

## Introduction

Skin and soft tissue infections (SSTI) are a preventable cause of morbidity and mortality among people who inject drugs (PWID) [1, 2]. In the United Kingdom, SSTI prevalence is rising [3], with hospitalisations for severe injecting-related infections increasing annually from 2012 [4]. Abscesses and cellulitis are common presentations, treatable with timely primary care [5]. Delayed care seeking can lead to serious infections such as septicaemia and endocarditis, requiring specialist inpatient intervention [6]. Costs associated with SSTI-related complications among PWID in the UK are substantial, with a 2012 estimate placing health care burden to the NHS in the region of £77 million per annum [7]. Studies have also documented outbreaks of invasive group A streptococcus (iGAS) and invasive Methicillin-Resistant Staphylococcus aureus (iMRSA) among street homeless PWID as well as prisoners and other marginalised populations. In the majority of these cases an injection site infection has been present [8, 9]. In a context of barriers to timely medical care access, PWID have been reported to self-medicate with old or incomplete antibiotic prescriptions [10, 11]. This is a concern, given potential for antiboitic resistance and vulnerability to MRSA aquistion [12].

A recent systematic review of injecting-related injury and disease found past-month prevalence of SSTI among PWID between 6% (UK) and 32% (USA), and lifetime prevalence between 6% (Australia) and 69% (Ireland) [13]. This variation is likely to relate to factors including drug type and form (e.g., ‘black tar’ heroin is associated with higher risk of SSTI than powder heroin), variable harm reduction and healthcare service provision, and drug preparation and administration practices [14–16]. UK National surveillance data found that 54% of PWID reported symptoms of an SSTI in the past 12 months, but did not capture severity or sequelae [3]. In this paper we present findings from an in-depth cross-sectional survey with PWID in London, with a focus on SSTI prevalence, severity, care seeking delay and associated health complications.

## Methods

Care & Prevent (C&P) is a mixed method project aiming to explore risk factors for, and prevalence of, SSTI and associated sequelae among PWID in London. Study rationale and methods detail have been published [17]. Ethical approvals were obtained from the London Bridge Research Ethics Committee [17/LO/0872] and the LSHTM Observational Research Ethics Committee [12021]. All participants provided written consent. Survey participants received a £10 voucher as reimbursement for their time.

### Participants

Participants were eligible for recruitment if they were aged 18 years or older at the time of the study and had a history of injecting psychoactive drugs. Recruitment was conducted through six drug treatment services and through a homeless outreach team visiting homeless hostels and day centres across London, UK. Participants were informed about the study through a notice in the service waiting room and through participant information sheets supplied by keyworkers and/or nurses during clinical appointments. Researchers were present at the sites on specified days to provide further information.

### Data generation

Data generation methods comprised: a computer assisted researcher administered SSTI risk factor questionnaire, urinalysis for proteinuria, and in-depth qualitative interviews. This paper reports findings from the questionnaire data, generated between October 2017 and March 2019 using Open Data Kit (ODK) software [18]. Data are available upon request.

### Measurements

**Fig 1:**
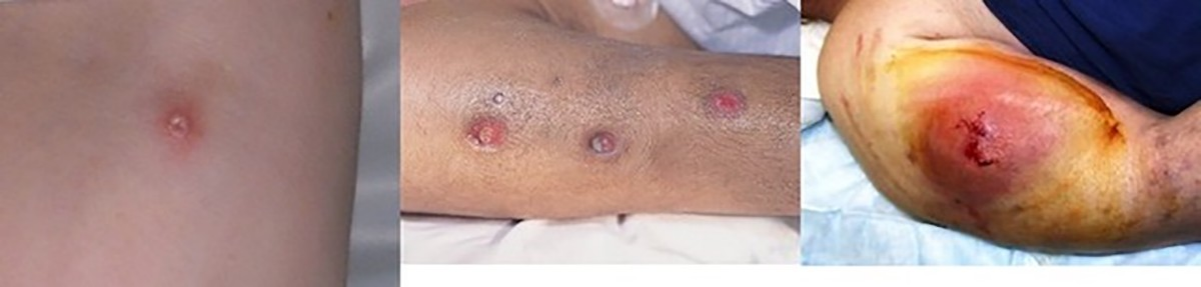
From left to right: Mild, moderate, and severe abscess.

**Fig 2:**
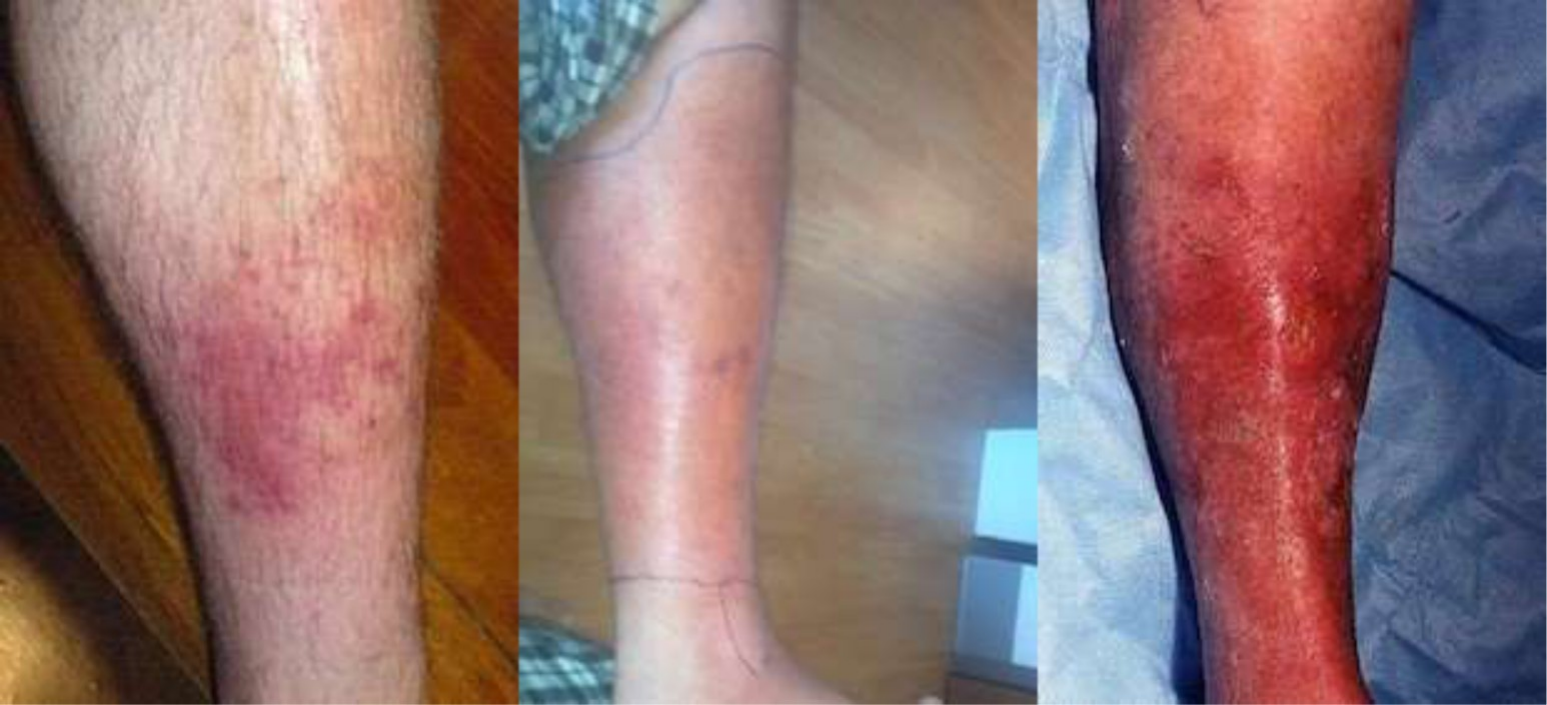
From left to right: Mild, moderate, and severe cellulitis.

The questionnaire was developed through consultation with a panel of experts and drew on existing surveys such as the Unlinked Anonymous Monitoring (UAM) Survey, a National surveillance study with PWID conducted annually [17]. Question domains comprised: socio-demographics; drug use history, injection preparation and administration practices (lifetime and previous 12 months); reuse and cleaning of injecting equipment (lifetime); experience of SSTI and other health conditions and care seeking practices (lifetime). Questions pertaining to SSTI experience were accompanied by pictures of abscesses and/or cellulitis at different stages (Figs 1 and 2). Still-based images are a reliable method for the assessment of wounds, their type and severity [19], with evidence that photographs can illicit clinical evaluations equivalent to those conducted in person [20]. Participants were shown SSTI photographs along with a verbal description of symptoms in order to help correct identification and to aid nomination of severity. For abscesses and cellulitis, participants were asked if they had experienced either infection, on how many occasions, bodily location/s and how they rated their worst infection (mild, moderate, or severe).

SSTI risk factor questions included detail on reuse of injecting equipment, injecting hygiene practices, primary drugs injected, type and amount of acidifier used to prepare injection solutions, number of injections per day, week or month, body sites injected and number of times taken to achieve an injection. The latter question was included as it provides a strong indication of peripheral venous damage–a risk factor for SSTI [16]. Participants were asked about care seeking practices for SSTI, including duration of time taken from noticing an infection to seeking medical advice and any SSTI-related hospitalisations. Measures pertaining to care seeking were informed by prior epidemiological SSTI risk factor and hospitalisation research [21], which used five or more days as an indicator of health care delay. We incorporated the five-day measure, also including ten or more days in recognition of the multiple barriers many PWID face in seeking care. Qualitative interviews provided specificity regarding time taken to seek care and the contexts which acted to facilitate or delay engagement with medical services. These findings are published separately [22].

In addition to SSTI and hospitalisation specific questions, participants were asked if they had received a medical diagnosis for the following health conditions: blood poisoning, arthritis, hepatitis B and C, deep vein thrombosis (DVT), diabetes, kidney disease, necrotising fasciitis, human immunodeficiency virus (HIV), chronic obstructive pulmonary disease (COPD), liver cirrhosis, tuberculosis, bone and/or joint infection, endocarditis, lymphangitis, and hypertension. These conditions were determined in relation to their potential to confound findings because they can cause inflammation, and/or affect the functioning of the immune or circulatory systems. We also asked participants whether they had ever had an invasive bacterial infection, defined as an infection of the blood, bones, joints, heart, or fascia. These infections were removed from the final analysis to demonstrate a clearer hypothetical causal pathway from comorbidities (other than invasive infections) to SSTIs to invasive infections.

### Statistical analysis

Descriptive statistics, including means, medians and ranges are presented for the study population, and those who reported lifetime history of an abscess and/or cellulitis. All variables in this study are reported across the participants’ injecting career, unless stated otherwise. Correlations between risk factors and having ever experienced an abscess and/or cellulitis were assessed using Chi-square tests of independence. Exposure variables associated with the outcome were assessed using logistic regression models on Stata version 15.1 [23]. Analyses were performed using logistic regression to assess those at higher risk of having an abscess and/or cellulitis. All regression models were adjusted using sex, age, and lifetime experience of street homelessness.

## Results

### Description of sample population

Four-hundred and fifty-five participants completed the questionnaire (Table 1). Three-quarters (75%) of the participants were men, with an average age of 46 years (IQR = 39–52). Over three quarters (78%) of the study population had lifetime history of street homelessness. SSTI prevalence was high, with 64% (95% CI 60–68) reporting lifetime experience of abscess and/or cellulitis. Of the 249 (55%) who reported at least one abscess, 90 (36%) rated their worst infection as severe, and 159 (64%) as mild or moderate. Among those who reported at least one cellulitis event in their lifetime, 34/155 (22%) rated their worst cellulitis as severe, and 121 (78%) as mild or moderate.

**Table 1:** Demographic distribution of those with an SSTI of any severity, and those with a severe infection with accompanying Pearson’s chi-square tests of independence.

Variable	Level	Total	Any SSTI	Χ^2^	p-value
		(N = 455)	(64%, n = 291)		
**Sex**	Male	341 (75.0%)	216 (63.3%)	0.22	0.64
	Female	114 (25.0%)	75 (65.8%)		
**Age**	<35 years old	58 (12.8%)	28 (48.3%)	20.04	<0.001
	35–44 years old	155 (34.1%)	77 (49.7%)		
	45+ years old	242 (53.2%)	186 (76.9%)		
**Ever homeless**	Yes	355 (78.0%)	231 (65.1%)	0.87	0.35
	No	100 (22.0%)	60 (60.0%)		
**Attempts (skin punctures) before achieving injection**	Once	202 (44.4%)	99 (49.0%)	41.26	<0.001
	Twice	82 (18.0%)	56 (68.3%)		
	Three times	58 (12.8%)	41 (70.7%)		
	Four or more times	108 (23.7%)	91 (84.3%)		
	Does not inject in vein	5 (1.1%)	4 (80.0%)		
**Injecting frequency**	Weekly (once to six times per week)	138 (30.3%)	31 (22.5%)	19.9	<0.001
	Daily or more	317 (69.7%)	260 (82.0%)		
**Injected in past 12 months**	Yes	284 (62.4%)	191 (67.3%)	3.56	0.06
	No	171 (37.6%)	100 (58.5%)		
**Main drug injected**	Heroin	199 (43.7%)	126 (63.3%)	18.60	0.10
	Crack	7 (1.5%)	1 (14.3%)		
	Heroin and crack combined	225 (49.5%)	155 (68.9%)		
	Methadone	5 (1.1%)	2 (40.0%)		
	Stimulants (Meth/amphetamines)	15 (3.3%)	6 (33.3%)		
	Cocaine	2 (0.4%)	1 (50.0%)		
	Methadrone	1 (0.2%)	-		
	Steroids	1 (0.2%)	-		
**Wash hands prior to injecting**	Always	134 (29.4%)	81 (60.4%)	1.01	0.60
	Sometimes	165 (36.3%)	108 (65.5%)		
	Never	156 (34.3%)	102 (65.4%)		
**Wipe injection site prior to injecting**	Always	207 (45.5%)	126 (60.9%)	1.59	0.45
	Sometimes	145 (31.9%)	96 (66.2%)		
	Never	103 (22.6%)	69 (67.0%)		
**Reuse needles or syringes**	Yes	306 (67.3%)	224 (73.2%)	34.66	<0.001
	No	149 (32.7%)	67 (45.0%)		
**Reuse filters**	Yes	190 (43.7%)	177 (93.2%)	10.52	0.001
	No	245 (56.3%)	109 (44.5%)		
**Comorbidity (diagnosed)**	No conditions	92 (20.2%)	34 (37.0%)	59.64	<0.001
	One condition	143 (31.4%)	81 (56.6%)		
	Two conditions	94 (20.7%)	71 (75.5%)		
	Three conditions	66 (14.5%)	52 (78.8%)		
	Four conditions	34 (7.5%)	30 (88.2%)		
	Five conditions or more	26 (5.7%)	23 (88.5%)		

Χ^2^ –Pearson’s chi-square test of independence.

p-value–significance at the 0.05 level or less.

In univariable analysis, SSTI was associated with older age, number of attempts before successful injection into a vein, injecting frequency per week, reusing needles and/or syringes, and reusing filters. Most participants with an abscess and/or cellulitis were aged 45 or older, injected once or more a day, required four or more attempts before successful injection into a vein, and reported reusing needles and/or syringes, and reusing filters.

**Fig 3:**
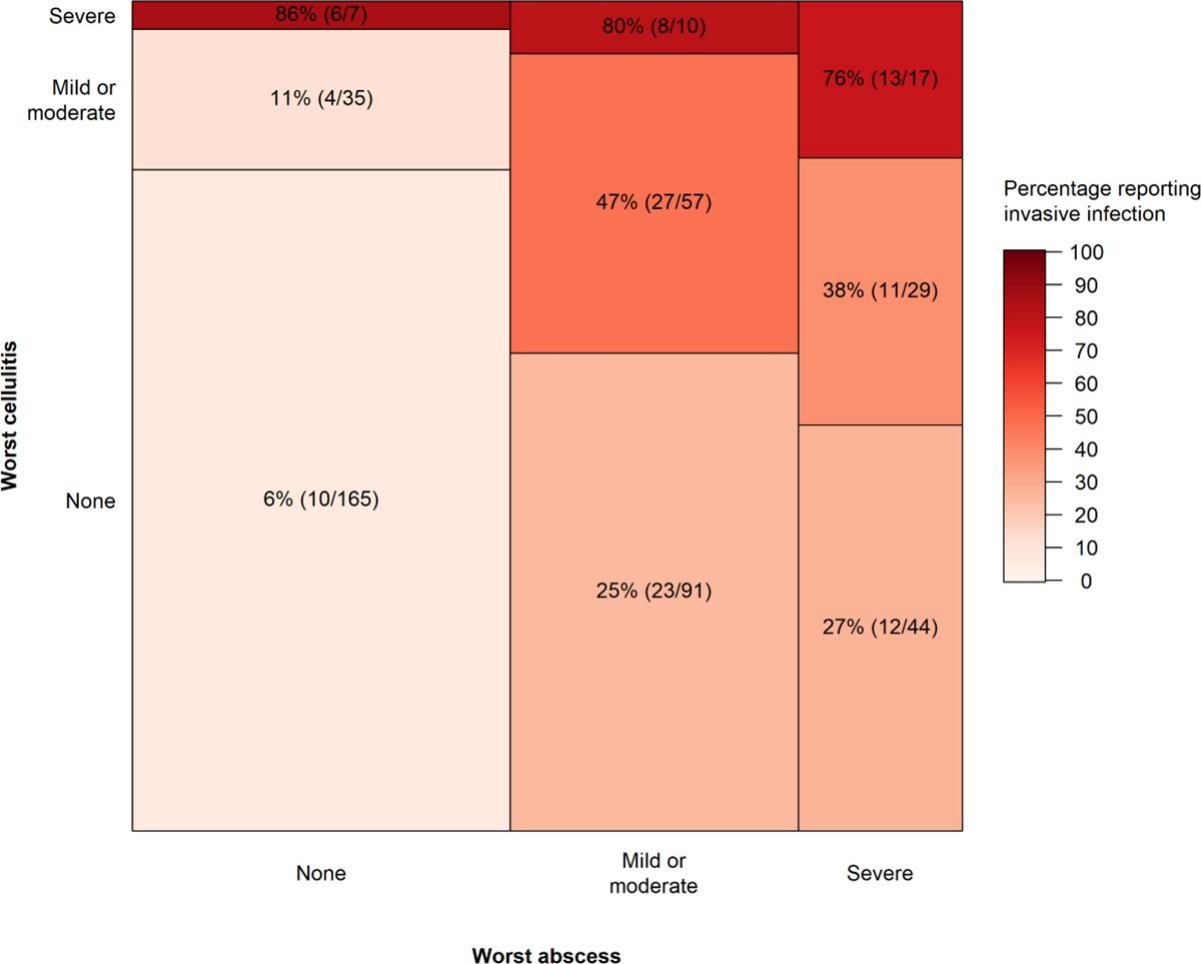
Number of participants reporting lifetime abscess and/or cellulitis by severity, and proportions reporting invasive infections (infections of the blood, bones, joints, heart, and necrotising fasciitis). The box size represents the number of participants with each combination of abscess/cellulitis severity, and the colour and percentage labels represent the proportion reporting invasive infections.

The relationship between abscesses or cellulitis and invasive infections (e.g. septicaemia and endocarditis) is summarised in Fig 3. Invasive disease is reported more often among those reporting severe cellulitis. It is also more frequent among those who have had any abscess than any cellulitis; however, when any cellulitis is co-reported with an abscess it greatly increases the likelihood of invasive disease.

The majority of participants employed self-care for their worst SSTI after first noticing symptoms (n = 135, 46%), followed by seeking medical care (n = 95, 33%). When asked how long they took to seek medical advice, 60% of participants (121) reported waiting for more than five days after first noticing symptoms, with more than a third with severe infections waiting for 10 or more days before seeking medical advice or not seeking advice at all (41, 43%). Of those who reported a bacterial infection, 137/291 (47%) reported an SSTI-related hospitalisation. The proportion reporting hospitalisation was higher among those with a severe infection, at 79/107 (74%).

### Factors associated with bacterial SSTIs

Lifetime SSTI was associated with being aged 35–44 or 45+ (compared to being aged under 35); injecting once or more a day; sometimes or always reusing needles, syringes, and/or filters; practicing sub-cutaneous or intra-muscular injection; and taking four or more attempts (skin punctures) to achieve an injection. Crude and adjusted associations are presented in Table 2. Compared with those reporting any SSTI, those who reported having had a severe bacterial infection had higher odds of taking ten or more days before seeking medical attention, after controlling for other SSTI risks [AOR 2.29 (95%CI 1.25–4.20).

**Table 2:** Univariate and bivariate regressions between SSTI bacterial infections and health outcomes. Adjusted models included sex, age, and lifetime experience of street homelessness.

		Ever had bacterial SSTI		
		(64%, n = 291)		
Variable	N	n(%)	Crude	Adjusted
			OR (95% CI)	AOR (95% CI)^+^
**Age**				
<35	58	28 (48.3%)	1	1
35–44	155	77 (49.7%)	1.92 (1.05–3.51)	2.03 (1.11–3.71)
45+	242	186 (76.9%)	3.32 (1.90–5.82)	3.68 (2.09–6.50)
**Injecting frequency**				
Less than once a day	138	31 (22.5%)	1	1
Once a day or more	317	260 (82.0%)	1.72 (1.14–2.59)	1.61 (1.05–2.45)
**Frequency of needle reuse**				
Never	149	67 (45.0%)	1	1
Sometimes	255	184 (72.2%)	3.17 (2.08–4.85)	3.44 (2.23–5.30)
Always	51	40 (78.4%)	4.45 (2.12–9.35)	4.04 (1.84–8.89)
**Frequency of filter reuse**				
Never	190	109 (57.4%)	1	1
Sometimes	156	108 (69.2%)	1.67 (1.07–2.61)	1.80 (1.14–2.84)
Always	89	69 (77.5%)	2.56 (1.44–4.56)	2.67 (1.48–4.81)
**Wash hands prior to injecting**				
Yes	299	189 (63.2%)	1	1
No	156	102 (65.4%)	1.10 (0.73–1.65)	1.14 (0.75–1.72)
**Wipe injection site prior to injecting**				
Yes	352	222 (63.1%)	1	1
No	103	69 (67.0%)	1.19 (0.75–1.89)	1.13 (0.70–1.82)
**Sub-cutaneous injecting (past 12 months)**				
No	110	62 (56.4%)	1	1
Yes	176	136 (77.3%)	2.63 (1.57–4.41)	2.55 (1.49–4.35)
**Intra-muscular injecting (past 12 months)**				
No	168	100 (60.0%)	1	1
Yes	118	98 (83.1%)	3.33 (1.88–5.90)	3.10 (1.74–5.52)
**Attempts before achieving injection**				
Once	202	99 (49.0%)	1	1
Twice	82	56 (68.3%)	2.24 (1.30–3.85)	2.14 (1.24–3.66)
Three times	58	41 (70.7%)	2.51 (1.34–4.71)	2.43 (1.32–4.50)
Four or more times	108	91 (84.3%)	5.57 (3.10–10.02)	5.01 (2.75–9.14)
Does not inject in vein	5	4 (80.0%)	4.16 (0.46–37.98)	3.01 (0.30–29.82)
**Years injecting main drug**				
One year or less	57	15 (26.3%)	1	1
2–4 years	70	31 (44.3%)	2.23 (1.05–4.74)	2.49 (1.16–5.34)
5–7 years	50	30 (60.0%)	4.20 (1.85–9.52)	3.95 (1.73–9.02)
8–10 years	55	35 (63.6%)	4.90 (2.19–10.98)	4.84 (2.14–10.92)
11–14 years	31	27 (87.1%)	18.90 (5.66–63.10)	21.84 (6.47–73.73)
15+ years	192	153 (79.7%)	10.98 (5.53–21.84)	10.05 (4.99–20.26)
**Comorbidity (diagnosed)**				
No conditions	92	34 (37.0%)	1	1
One condition	143	81 (56.6%)	2.23 (1.30–3.82)	2.01 (1.16–3.49)
Two conditions	94	71 (75.5%)	5.27 (2.80–9.92)	4.88 (2.57–9.26)
Three conditions	66	52 (78.8%)	6.34 (3.06–13.11)	5.48 (2.62–11.43)
Four conditions	34	30 (88.2%)	12.79 (4.14–39.42)	10.50 (3.39–32.51)
Five conditions or more	26	23 (88.5%)	13.08 (3.65–46.89)	10.02 (2.67–37.57)

Reported comorbidities, other than invasive infections, were common among those who have experienced a SSTI. Half (48%, n = 220) of the sample reported at least two or more diagnoses of health conditions. Those who reported having one or multiple health conditions were at higher odds of co-reporting a SSTI. There was a strong, graded association between the number of comorbidities and the likelihood of reporting a lifetime SSTI.

## Discussion

We used a novel survey technique using photographs of injecting related wounds to measure prevalence, type and severity of bacterial SSTIs among a sample of people who inject drugs in London. Two-thirds of participants reported ever having had an abscess and/or cellulitis, with many prioritising self-care and/or taking more than five days to engage with medical care after first noticing an infection. Nearly half of those experiencing an SSTI reported a serious infection requiring hospitalisation. Invasive bacterial diseases were more commonly reported by those who had also reported a bacterial SSTI, particularly those with severe localised infections, suggesting that safer injecting and timely access to wound care in the community may help prevent serious infections.

Our findings on the risks of injection frequency, and non-venous injections e.g. sub-cutaneous and intra-muscular, confirm those found by others [20, 24]. A new finding is that of number of skin punctures per injection is also associated with the risk of SSTI. In our results, four or more injection attempts prior to successful injection was independently associated with SSTI and severe SSTI. This may reflect loss of peripheral veins due to venous sclerosis, which may be due to overuse of acidifiers in injection preparation [25]. As reported in a related study publication [16], overuse of citric acid or vitamin C in injection preparation can exacerbate venous sclerosis. This in turn can require transitions to more risky injection sites (such as the legs and groin) as well as to increase the likelihood of accidental or purposeful non-venous injections and associated SSTI risk.

We found a strong graded association between co-morbid health conditions and the risk of SSTI. This reflects a number of causal pathways, probably acting in both directions. For example, disease such as diabetes and HIV may reduce immunity and increase susceptibility to SSTI. On the other hand, inflammation associated with repeated SSTI may increase the risk of diseases such as arthritis and kidney disease [26]. In the case of septicaemia and endocarditis, untreated bacterial infections can spread, through the blood, from the skin infection site causing serious systemic infection [5]

We highlight an association between severity of infection and the time taken to seek healthcare. We found that most (60%) of our cohort indicated taking five or more days to seek medical attention after first noticing the symptoms of a bacterial SSTI. This duration has been previously used as a measure of care delay [21] and reflects NHS guidance that care should be sought immediately on noticing symptoms of a bacterial infection [27, 28]. In adjusted analysis, seeking healthcare 10 or more days after noticing symptoms was independently associated with ever having a severe SSTI. Notable was the high rates of reported hospitalisation (47%, n = 137) associated with preventable bacterial infections. Given these findings we posit that duration of time in seeking healthcare impacts severity of bacterial SSTI. Taking ten or more days to seek healthcare for an SSTI appears to enhance risk of health complications, such as septicaemia, and associated inpatient hospital admission.

It is well evidenced that PWID face significant barriers in accessing appropriate healthcare due to stigma and discrimination from healthcare professionals as well as structural difficulties in accessing and attending medical appointments [29–31]. Qualitative participant data, reported elsewhere [22] illustrate that delays in seeking medical care are informed by a complex interplay of social structural issues, including limited time due to money generation demands; limited hope and expectation of adequate care; fear of opiate withdrawal in the hospital system; and normalised pain in a context of pervasive everyday violence. We emphasise therefore, the resilience demonstrated when seeking care in constrained circumstances and the need for structural, rather than purely educational, interventions in capacitating change [22]. Nearly half (46%) of participants with an SSTI reported undertaking self-care as the first response to their worst infection. Self-care for bacterial infections is generally not recommended, with some practices (such as self-lancing of abscesses) potentially acting to exacerbate infection. In a context of barriers to health care, self-care will continue to be the preferred option for many. Support and resourcing to enable PWID to care for infections (such as wound care training and dressings) can help facilitate safer practices but also trust and engagement with providers, enhancing healthcare access opportunity.

The proportion of participants reporting a lifetime SSTI in our results (64%) is at the high end of a range reported in a recent systematic review [13]. This may be related to risk factors in our sample, such as homelessness or the age of participants, or the method we used, which included photos to help participants identify infections. 30% of participants reported being hospitalised for an SSTI at some point in their life. In a recent cohort study of PWID in opiate substitution treatment in South London, 24% of participants were hospitalised at least once for a bacterial infection over an average of eight years’ follow-up [32]. These broadly similar values suggest that similar populations were captured by these two research methods, and also show that the majority of SSTI are treated outside of hospital.

### Strengths and weaknesses

This study captured a wide range of injecting-related risk factors and measured the severity of infections, which to our knowledge has not been measured in previous studies. The survey method allowed us to study detailed aspects of participants’ risk environment. The cross-sectional design meant that we were not able to establish a temporal relationship between bacterial SSTIs and these injecting risk factors, or report incidence. This contrasts with longitudinal studies using healthcare records, which provide more precise estimates of incidence but often lack detailed risk factor information [32] Self-reported health conditions reported are susceptible to problems with recall and awareness of diseases, such as kidney disease, where participants may have been informed of a urinary tract infection but misconstrued this as kidney disease, and/or may not be aware if the disease is acute or chronic, particularly if they are not currently engaging with healthcare services.

This study is of importance due to its use of photographs to elicit SSTI recall, which contextualises and increases our understanding of risk factors. The validity of the approach of still based images for self-report of SSTI severity is not known. The images were chosen in consultation with skin infection specialists, but have not been validated or compared to a gold standard (such as clinician evaluation). Despite this, there is plausible predictive validity in the sense that those with more severe local infections (abscesses and cellulitis) had higher prevalence of invasive infections. Clinical diagnosis was not possible in this study as recruitment was opportunistic and access to skin infection specialist clinicians was not available.

Recruitment through community rather than hospital settings captures a more thorough assessment of prevalence by engaging those who have restricted access to hospitals and services. Analysis has demonstrated that C&P sample is comparable to those of PWID participating in the larger National UAM surveillance survey [32]. We have some confidence, therefore, that findings reported in this paper are generalisable to the broader population of PWID in the UK [33]

## Conclusion

We found high lifetime prevalence of SSTI among PWID in London, with severity and health complications potentially exacerbated by delayed medical care seeking. Interventions to reduce SSTI prevalence, severity and sequalae need to attend both to injection practice risk factors but also the social structural barriers faced by PWID in accessing timely health care.

## Supporting information

S1 Data (XLSX)
